# Reply: Regarding Does a Composite Plaque Phenotype Capture Vulnerability or Atherosclerotic Burden?

**DOI:** 10.1016/j.jacbts.2026.101748

**Published:** 2026-10-28

**Authors:** Anish Karpurapu, Pamela S. Douglas, Borek Foldyna, Svati H. Shah

**Affiliations:** a Duke University School of Medicine, Durham, North Carolina, USA; b Division of Cardiology, Department of Medicine, Duke University School of Medicine, Durham, North Carolina, USA; c Duke Clinical Research Institute, Duke University School of Medicine, Durham, North Carolina, USA; d Department of Radiology, Massachusetts General Hospital, Boston, Massachusetts, USA; e Duke Molecular Physiology Institute, Duke University School of Medicine, Durham, North Carolina, USA

We thank Drs İşleyen and Bulut for their thoughtful comments regarding the interpretation of our findings. Our work highlighted proteins associated with a high-risk composite phenotype (HRCP), defined by the presence of obstructive coronary artery disease (CAD), a computed tomography–adapted Leaman score >5, coronary calcium >400, or at least 1 high-risk plaque (HRP) feature.^1^ We used this composite to capture high-risk coronary atherosclerosis at the patient level, incorporating complementary measures of disease including plaque extent, stenosis severity, calcification, plaque composition, and adverse plaque morphology to estimate total anatomic risk.

We agree that analyses of individual HRCP features demonstrated that the Leaman score was the primary driver of observed protein associations. However, we would like to emphasize that the computed tomography–adapted Leaman score is not simply a measure of plaque extent. In addition to the amount of coronary atherosclerosis, it incorporates information on plaque location, stenosis severity, and plaque composition and therefore provides a more granular assessment of CAD than simpler measures of plaque burden, such as the segment involvement score.

As such, we agree with the authors’ comment that the identified protein signatures should not be interpreted as a molecular signature specific to plaque vulnerability independent of overall atherosclerotic burden. Our exploratory HRP-specific analyses support this distinction, as only 1 protein was associated with HRP after multivariable adjustment, and none remained significant after multivariable adjustment when analyses were restricted to individuals without obstructive CAD. As delineated in the discussion, these findings do not establish a signature specific to plaque vulnerability independent of burden but are instead consistent with the broader HRCP phenotype we intended to study.

The proposed subgroup analyses comparing mutually exclusive combinations of HRP and Leaman score are scientifically interesting but further subdivide an already limited HRP population and further limit statistical power. Moreover, the HRCP components substantially overlap and are likely manifestations of the same underlying coronary atherosclerotic process, which would limit the interpretation of mutually adjusted analyses.
